# Fertility intentions and contraceptive practices among clinic-users living with HIV in Kenya: a mixed methods study

**DOI:** 10.1186/s12889-017-4514-2

**Published:** 2017-07-05

**Authors:** Susannah H. Mayhew, Manuela Colombini, James Kelly Kimani, Keith Tomlin, Charlotte E. Warren, for the Integra Initiative, Richard Mutemwa

**Affiliations:** 1 Department of Global Health and Development, London School of Hygiene & Tropical Medicine, Keppel St, London, WC1E 7HT UK; 2 Department of Epidemiology and Population Health, London School of Hygiene & Tropical Medicine, London, WC1E 7HT UK; 3 Department for International Development (DfID), (at the time of this research, Kimani was with the Population Council, Nairobi), Nairobi, Kenya; 4 Population Council, Washington, DC USA; 5 Centre for Infectious Disease Research (at the time of this research, Mutemwa was with the LSHTM), Lusaka, Zambia

**Keywords:** Fertility, Contraception, Women living with HIV, Africa, Mixed methods

## Abstract

**Background:**

Preventing unwanted pregnancies in Women Living with HIV (WLHIV) is a recognised HIV-prevention strategy. This study explores the fertility intentions and contraceptive practices of WLHIV using services in Kenya.

**Methods:**

Two hundred forty women self-identifying as WLHIV who attended reproductive health services in Kenya were interviewed with a structured questionnaire in 2011; 48 were also interviewed in-depth. STATA SE/13.1, Nvivo 8 and thematic analysis were used.

**Results:**

Seventy one percent participants did not want another child; this was associated with having at least two living children and being the bread-winner. FP use was high (92%) but so were unintended pregnancies (40%) while living with HIV. 56 women reported becoming pregnant “while using FP”: all were using condoms or short-term methods. Only 16% participants used effective long-acting reversible contraceptives or permanent methods (LARC-PM). Being older than 25 years and separated, widowed or divorced were significant predictors of long-term method use. Qualitative data revealed strong motivation among WLHIV to plan or prevent pregnancies to avoid negative health consequences. Few participants received good information about contraceptive choices.

**Conclusions:**

WLHIV need better access to FP advice and a wider range of contraceptives including LARC to enable informed choices that will protect their fertility intentions, ensure planned pregnancies and promote safe child-bearing.

**Trial registration:**

Integra is a non-randomised pre-post intervention trial registered with Current Controlled Trials ID: NCT01694862.

## Background

With improved treatment and survival rates for HIV the disease is becoming a chronic disease requiring management through the life-course. Women Living with HIV (WLHIV) can now expect a near-normal reproductive life and studies from sub-Saharan Africa show that many WLHIV desire children in the near future, despite their status [1–5]. Nevertheless, compared to HIV-negative women, evidence generally indicates that fertility intentions are lower among WLHIV [6–10]. A wide range of studies across sub-Saharan Africa show that HIV status either decreases the overall desire for more children or increases the period of delay before the next child [1, 6, 10–13]. Related to this, other studies show continuing high rates of unintended pregnancies (i.e. pregnancies that are either unwanted or mistimed) among WLHIV in Sub-Saharan Africa [10, 14–17]. Safe childbearing is increasingly possible even for WLHIV from deprived communities. To achieve this, however, pregnancies must be planned to avoid high risk of vertical transmission as well as possible negative consequences of unplanned pregnancy on disease progression [18–20]. This is why adequate contraception for WLHIV is a core component (Prong 2) of the WHO/UNAIDS HIV prevention strategy [21]. Despite this, many studies indicate a high unmet need for contraception among WLHIV in Sub-Saharan Africa [7, 8, 22, 23] although evidence is somewhat mixed. The predominance of the use of condoms as a contraceptive method by WLHIV is well documented [7, 9, 13] and the use of short-term methods has been associated with unplanned pregnancies, for example among WLHIV in Swaziland [24].

Like many countries the prevalence of HIV in Kenya is higher among women (8%) than men (4%) [25]. In Central Province 6.2% women aged 15–49 are infected while in Eastern Province it is 3.8% women [25]. There are few studies specific to Kenya on fertility intentions of WLHIV and this is not reported in Demographic and Health Surveys or in UNAIDS Country reports. One small qualitative study in Kenya found a high unmet need for contraception among WLHIV in Kenya, who used mainly condoms for contraception [22]. Another study in Kenya found that 54% WLHIV report their last childbirth was unintended and of these only 26% were using modern contraception, compared to 46% in the HIV-negative population [26]. One population-based study in Kenya and Swaziland found there were missed opportunities in both countries for meeting dual needs of women for family planning and HIV services among clinic users [27]. Analysis of Integra data from a cohort of WLHIV over time in Kenya showed that, despite good use of contraception by WLHIV, the proportion of mistimed and unintended pregnancies was higher among HIV-positive women compared to HIV-negative women [10]. This suggested problems using contraception or inconsistent use. Male attitudes are known to cause significant problems for some women wishing to use contraception. A qualitative study in Western Kenya identified male fears that contraception would increase women’s sexual agency and promiscuity and threaten male roles as breadwinner [28].

The objective of this present study was to explore the fertility desires and contraceptive practices of WLHIV who are users of reproductive health services in Kenya, with a view to understanding how services are meeting the needs of existing clients and whether they can be improved.

## Methods

The Integra Initiative is a longitudinal non-randomised mixed-method evaluation of integrated models of reproductive health-HIV service delivery in 40 clinics in Kenya and Swaziland [29]. The study employed a controlled pre- and post-test quasi-experimental, or non-randomised, design. Before recruitment of participants, providers in study intervention facilities were trained in provision of integrated services using a Balanced Counselling Strategy Plus algorithm (BCS+) and a standardised mentorship strategy described elsewhere [29]. Study implementation begun after intervention-facility providers were certified as attaining a pre-determined minimum level of clinical skills. However, the effects of the intervention were shown to have no effect because of “real world” design in which researchers had no control over clinic organisation, staff turnover and migration between sites, external donor activities and evolving policy which meant that over time some control sites also integrated services during the study [30]. A new, clinic-specific, measure of “integration” was therefore developed to assess the impact on health outcomes [30].

This paper draws on the Integra Initiative’s cohort study to explore the fertility intentions and contraceptive practices of WLHIV using services in Kenya, regardless of whether they were using an integrated clinic or not. Participant’s experiences of integration have been reported elsewhere [31]. The cohort data involved 2291 clients recruited at 24 public-sector family planning (FP) and post-natal care (PNC) facilities in Kenya followed for 24 months between October 2009–April 2010 and October 2011–April 2012. This cohort includes a sub-sample of 240 women who self-identified as living with HIV. This sample is the focus of this paper. A total of four quantitative questionnaires were completed over the 24 months. Eligibility criteria for the survey were: being aged 18 years or more, being a clinic user (either a repeat FP user or a post-natal care user attending between 0 and 10 weeks of a birth), be living in the catchment area of the health facility, and willing to give their informed consent to be interviewed. Additionally for this analysis we included only women who self-reported their HIV positive status. Forty-eight of these women additionally participated in two rounds of qualitative in-depth interviews.

Longitudinal results are reported elsewhere [10]. The data reported here are from the third round of survey questionnaires which was analysed cross-sectionally together with the first set of linked in-depth interviews which occurred around the same time, to allow in-depth analysis of fertility desires and contraceptive practices.

Both the survey and in-depth interviews took place approximately 15 months after recruitment – this means that the “PNC” clients were no longer PNC clients but had become FP clients or HIV clients. For the purposes of this paper, to avoid misleading labels, we refer to these cohorts by the Provinces in which they were recruited: Central Province (FP facilities) and Eastern Province (PNC facilities). We differentiate between the cohorts in the analysis to indicate their point of recruitment and highlight any observable differences over time.

Apart from the recruitment interview, which was at the health facility, all follow-up survey and qualitative interviews were conducted outside the facilities, unless explicitly requested by the interviewee. This minimised service-related courtesy bias and helped women feel more comfortable sharing their perceptions, even when they differed from clinical advice.

### Quantitative data

Consecutive sampling of female clients was undertaken at study facilities 24 of which had clients self-reporting HIV. 204 clients attending FP services in Central Province and 36 clients attending PNC services in Eastern Province were recruited. A total of 240 women self-reporting to be living with HIV were interviewed in Kiswahili between February and July 2011. The smaller number recruited at PNC facilities reflects the smaller number of WLHIV attending those facilities (for the rarer event of childbirth) compared to those attending family planning and the somewhat lower HIV prevalence in Eastern Province compared to Central Province. The survey data were collected by project-trained interviewers using Personal Digital Assistant devices.

#### Statistical analysis

All statistical analyses were conducted in STATA SE/13.1. Socio-demographic and family planning behaviour factors were selected based on literature and observational experience.

Univariate tests of the socio-demographic factors were conducted using chi-squared and Fisher’s exact tests (accordingly) to assess distributional differences across the sub-cohorts. Logistic regression analysis focused on two behavioural binary outcomes: fertility desires and type of family planning method used among women living with HIV/AIDS. “Fertility desire” was represented by whether a woman ever wanted to have another child, or did not want to. Type of modern family planning method used was presented as long-term methods (sterilisation, implants, IUDs), or short-term methods (all others).

For fertility desire, chi-square tests and Fisher’s exact tests, accordingly, were used to assess association between socio-demographic/ family planning factors and the woman’s propensity to want another child. All the factors that demonstrated significant (α = 0.05) univariate association with the woman’s propensity to want another child were subsequently loaded in a multivariable logit model, with the woman’s propensity to want another child as the outcome. Finally, a predictive model for the woman’s propensity to want another child was constructed using stepwise forward selection for choosing predictor covariates used in the fitted logit regression model. Only those covariates that added meaningful information to the predictive model were selected. The variable “*parity balance*” was constructed from the difference between “*number of living children*” and “*desired number of children*”. On fitting the predictive model it was felt that the original 2 variables on which data were actually collected would be more useful, thus, “*number of living children*” and “*desired number of children*” were fitted in the predictive model and assessed. It was anticipated that the strength of association between fertility desire and “*number of living children*” and “*desired number of children*” would change, after the exclusion of “*parity balance*” from the predictive model; this did happen as shown in the modelling results below. The Bayesian Information Criterion for each additional covariate was estimated and selection rules applied as recommended by Raftery [32] in deciding whether to include or exclude a covariate. To determine the relative influence of the selected covariates in the predictive model, fully standardized coefficients were estimated for the covariates using the method described by Pampel [33] and Long [34]. These analysis steps were repeated for the outcome of type of family planning method used.

### Qualitative data

Many of the 240 women gave their consent to be followed up for in-depth interviewing and we attempted to trace 100 women for whom we had valid telephone numbers and were purposively selected to ensure representation from a wide range of study facilities. In total 48 participants were interviewed: 25 from Central Province (from 9 of the 12 study facilities) and 23 from Eastern Province (from 9 of 12 facilities). Where no participants were interviewed from a facility this was because no WLHIV from that facility could be recruited either because there were no WLHIV attending that facility (one facility) or a selected WLHIV declined consent when asked for an in-depth interview (10 in Central and none in Eastern), could not be traced (31 in Central, 8 in Eastern) or had died (2 in Central, 1 in Eastern).

The purpose of this planned qualitative component was to: explore participants’ views on fertility intentions; explore the nature of contraceptive use among WLHIV; and explore the medical advice received at linked services on pregnancy prevention. A topic guide was developed from literature on fertility intentions and FP practices among WLHIV, some preliminary analysis of the round 1 quantitative survey data and the core Integra objectives. This guide was pre-tested in 2011 among 8 participants at one (non-study) clinic. Subsequently, it was refined and eight local interviewers were given a four-day training (by Integra research partners) on qualitative data collection, the topic guide and interview practice and critique.

Face-to-face interviews were then conducted in Kiswahili between January and March 2011. The selected participants (from the quantitative survey) were asked to reconfirm their consent for an in-depth interview. Interviews were conducted in private locations chosen by the interviewees. Most took place in the participants’ homes; none took place in health facilities. The interviews took approximately one hour to complete. All interviews were audio-recorded, transcribed then translated into English.

The transcripts were coded by two of the authors (SHM and MC), who have worked together extensively, using NVivo 8.0 and analysed using thematic analysis. They cross-checked a sample of their coding of transcripts to ensure consistency. The core themes were derived deductively using topics covered in the interview, including fertility intentions, experiences of using FP, planning of pregnancies and whether HIV status affected any of these. Subsequently, emerging themes and discrepancies across them were discussed with co-authors and local partners to ensure validity of the findings. A coding hierarchy was adjusted and refined during analysis, until overarching themes were identified. Coded text from core themes was then transferred from the soft-ware package to excel sheets for detailed inductive analysis of experiences, views and other emerging issues within each theme. Each of these sub-themes is reported under the core themes in the qualitative results below.

## Results

### Description of quantitative sample

There were 204 WLHIV in Central Province and 36 WLHIV in Eastern Province; differences between them are shown Table 1. The Eastern cohort (who had all given birth around 18 months previously, after becoming HIV+) were more likely to be younger, married, unemployed, have a partner who was the breadwinner, not be using family planning (although 73% were) and not be using dual protection (although 45% were) when compared to Central cohort. Eastern cohort were also more likely to have fulfilled their desired number of children (with the last birth) and more likely to have no currently active sexual partners (22% in the last month compared to 0% Central clients). Finally, perhaps surprisingly, Eastern (PNC recruited) cohort were less likely to be on ART (53% compared to 79% Central clients). This may be an artefact of this cohort being slightly younger than the Central cohort participants and possibly having had a shorter time since diagnosis meaning they had not yet reached a CD4 threshold at which ART would be necessary (although we lack time-since-diagnosis data to verify this). All these differences were controlled for in subsequent analyses where necessary.

**Table 1:** Main socio-demographic, fertility and family planning variables by study cohort for the Quantitative sample (*N* = 240)

	Study cohort		
Factor	Central Province *N* = 204 (%)	Eastern Province *N* = 36 (%)	*χ* ^2^/Fisher’s *p-value*
Age			
16–25	15 (7)	8 (22)	*<0.001*
26–35	114 (56)	26 (72)	
36–45	75 (37)	2 (6)	
Marital status			
Single	75 (37)	4 (11)	*0.003*
Married	129 (63)	32 (89)	
Religion			
Roman Catholic	58 (28)	13 (36)	*0.352*
Other	146 (72)	23 (64)	
Education			
Up to primary	169 (83)	29 (81)	*0.739*
Above primary	35 (17)	7 (19)	
Employment			
Unemployed	50 (24)	26 (72)	*<0.001*
Regular	145 (72)	9 (25)	
Professional	9 (4)	1 (3)	
Breadwinner status			
Herself	76 (37)	3 (8)	*0.001*
Partner	114 (56)	29 (81)	
Other	14 (7)	4 (11)	
Breadwinner occupation			
Unemployed	8 (4)	0 (0)	*0.739*
Regular	178 (87)	33 (92)	
Professional	18 (9)	3 (8)	
Has health insurance			
No	171 (84)	31 (86)	*0.729*
Yes	33 (16)	5 (14)	
Household income			
< KSh3000	46 (23)	13 (36)	*0.216*
3000–9999	113 (55)	16 (44)	
10,000+	45 (22)	7 (19)	
Can raise KSh1000 for emergency			
Easy to	38 (19)	7 (19)	*0.908*
Difficult to	166 (81)	29 (81)	
Can access FP voucher			
No	182 (91)	27 (90)	*0.732*
Yes	17 (9)	3 (10)	
Currently on ART			
No	42 (21)	17 (47)	*0.001*
Yes	162 (79)	19 (53)	
On Co-trimoxazole prophylaxis			
No	22 (11)	7 (19)	*0.164*
Yes	182 (89)	29 (81)	
Knowledge of partner’s HIV status			
No	55 (28)	9 (28)	*0.112*
Yes	145 (72)	23 (72)	
Ever experienced stigma			
No	146 (72)	33 (92)	*0.011*
Yes	58 (28)	3 (8)	
Sexually active			
No	55 (28)	11 (37)	*0.135*
Yes	144 (72)	19 (63)	
Number of sex partners in last 1 month			
0	0 (0)	7 (22)	*<0.001*
1	116 (97)	25 (78)	
> 1	3 (3)	0 (0)	
Number of sex partners in last 12 months			
0	0 (0)	5 (16)	*<0.001*
1	131 (97)	26 (81)	
> 1	4 (3)	1 (3)	
Fertility Variables			
Number of previous pregnancies			
Once	26 (13)	1 (3)	*0.173*
Two times	69 (34)	10 (28)	
Three times	47 (23)	9 (25)	
More than three times	62 (30)	16 (44)	
Number of living children			
1 child	34 (17)	1 (3)	*0.007*
2 children	75 (38)	11 (31)	
3 children	53 (27)	9 (25)	
> 3 children	37 (19)	15 (42)	
Desired number of children			
2 children	66 (32)	6 (20)	*0.051*
3 children	75 (37)	8 (27)	
> 3 children	63 (31)	16 (53)	
Partner desires same number children			
No	23 (12)	4 (13)	*0.674*
Yes	101 (51)	18 (60)	
Has no partner	45 (23)	4 (13)	
Don’t know	30 (15)	4 (13)	
Parity balance			
More children than number desired	31 (15)	5 (14)	*0.228*
Desired number of children fulfilled	72 (35)	18 (50)	
Less children than number desired	101 (50)	13 (36)	
Wants another child			
No	143 (70)	24 (80)	*0.263*
Yes	61 (30)	6 (20)	
Intended the last pregnancy			
No	26 (13)	5 (14)	*0.901*
Wanted to wait	58 (28)	9 (25)	
Yes	120 (59)	22 (61)	
Feeling if unexpectedly pregnant today			
I would feel sad	118 (60)	22 (65)	*0.276*
I would feel happy	17 (9)	5 (15)	
Indifferent	62 (31)	7 (20)	
Family Planning variables			
Uses family planning			
No	10 (5)	8 (27)	*0.001*
Yes	189 (95)	22 (73)	
Type of family planning method used			
Short-term	97 (51)	10 (45)	*0.311*
Long-term	27 (14)	6 (27)	
Condom	65 (34)	6 (27)	
Dual protection			
No	61 (32)	12 (55)	*0.038*
Yes	128 (68)	10 (45)	
Provider explained FP methods			
No	113 (58)	9 (69)	*0.296*
Yes	82 (42)	4 (31)	
Stopped family planning			
Not stopped	168 (84)	28 (78)	*0.324*
Stopped	31 (16)	8 (22)	
Pregnant at baseline or over follow-up			
No	1 (0.5)	0 (0)	*1.000*
Yes	197 (99.5)	36 (100)	
Got pregnant while on Family Planning			
No	150 (78)	22 (63)	*0.060*
Yes	43 (22)	13 (37)	
FP method in use when she got pregnant			
Short-term & condoms	43 (100)	13 (100)	*−*
Long-term & permanent	0 (0)	0 (0)	

Missing data in some sub-categories means N is not always equal to 240 for each variable

### Influences on fertility desires among WLHIV

Table 1 shows descriptive data for fertility desires. The majority of women were sexually active (72% Central; 63% Eastern) and using FP (95% Central; 73% Eastern). Most (70% Central; 80% Eastern) did *not* want another child; 40% had experienced an unintended pregnancy last time they were pregnant, which was after they knew they were living with HIV. Two-thirds (60% Central; 65% Eastern) said they would feel sad if they became unexpectedly pregnant now.

Analysis showed that nine of the variables in Table 1 showed a univariable association with not wanting another child: see Table 2. Most associations were expected although education is notable since it shows nearly half of women with above-primary education want another child while only a quarter of those with lower education wanted another. After adjusting for confounding factors, only three variables showed significance: women who were breadwinners themselves and women who had no partner or their partner shared the same desired number of children, had lowest odds of wanting another child. Women who had fewer children than they desired had almost 7 times the odds of *wanting* another child compared to those who had more children than desired.

**Table 2:** Factors significantly associated with fertility desires among WLHIV in Kenya

	Fertility desire			
Factor	Did not want another child, *N* = 167 (%)	Wanted another child, *N* = 67 (%)	*χ* ^2^ *p-value*	AOR [95% CI]
Group				
Central Province	143 (70)	61 (30)	*0.263*	
Eastern Province	24 (80)	6 (20)		
Education				
Up to primary	145 (75)	49 (25)	*0.012*	1.00
Above primary	22 (55)	18 (45)		0.84 [0.27, 2.62]
Breadwinner status				
Herself	64 (81)	15 (19)	*0.007*	1.00
Partner	96 (61)	43 (39)		2.69 [0.92, 7.89]
Other	7 (44)	9 (56)		19.21 [2.81, 131.33]*
Breadwinner occupation				
Unemployed & Regular	161 (75)	53 (25)	*<0.001*	1.00
Professional	6 (30)	14 (70)		2.59 [0.54, 12.46]
Has health insurance				
No	148 (75)	49 (25)	*0.003*	1.00
Yes	19 (51)	18 (49)		2.03 [0.73, 5.69]
Can raise KSh1000 for emergency				
Easy to	24 (56)	19 (44)	*0.013*	1.00
Difficult to	143 (75)	48 (25)		0.68 [0.24, 1.90]
Number of living children				
1 child	10 (29)	25 (71)	*<0.001*	1.00
2 children	55 (66)	28 (34)		0.30 [0.07, 1.25]
3 children	51 (84)	10 (16)		0.37 [0.07, 1.98]
> 3 children	47 (94)	3 (4)		0.17 [0.02, 1.78]
Partner desires same number children				
No	15 (56)	12 (44)	*0.001*	1.00
Yes	88 (74)	31 (26)		0.41 [0.13, 1.31]
Has no partner	43 (88)	6 (12)		0.15 [0.03, 0.75]**
Don’t know	17 (50)	17 (50)		0.80 [0.21, 3.06]
Parity balance				
More children than number desired	33 (92)	3 (8)	*<0.001*	1.00
Desired number of children fulfilled	82 (91)	8 (9)		0.70 [0.14, 3.49]
Less children than number desired	52 (48)	56 (52)		6.33 [1.17, 34.37]†
Number of previous pregnancies				
Once	10 (37)	17 (63)	*<0.001*	1.00
Two times	46 (61)	30 (39)		2.41 [0.44, 13.36]
Three times	45 (82)	10 (18)		0.78 [0.12, 4.89]
More than three times	66 (87)	10 (13)		1.20 [0.17, 8.58]

*p = 0.002; **p = 0.021 † *p* = 0.032; some percentages may not add up to exactly 100% due to rounding; AOR = adjusted odds ratio

Table 3 presents the results from the multivariable predictive logit model. Analysis shows very strong evidence (*p* < 0.001), after controlling for the other factors, that “wanting another child” reduces as the number of living children the woman has increases. Women who are breadwinners remains significant for not wanting another child. The evidence for desired number of children and partner concordance on desired number of children appears to be somewhat mixed.

**Table 3:** Most significant predictors for wanting another child among WLHIV in Kenya

Factor	AOR	Standard error	[95% CI]	Standardized Beta
Breadwinner status				
Herself	1.00	-	-	-
Partner	2.33	1.175	[0.87, 6.26]^†^	0.17
Other	15.53	14.110	[2.62, 92.17]^**^	0.27
Number of living children				
1 child	1.00	-	-	-
2 children	0.15	0.080	[0.05, 0.43]^*^	−0.36
3 children	0.04	0.026	[0.01, 0.14]^*^	−0.56
> 3 children	0.01	0.010	[0.002, 0.05]^*^	−0.73
Desired number of children				
>3 children	1.00	-	-	-
2 children	0.26	0.135	[0.09, 0.72]^§^	−0.25
3 children	0.90	0.403	[0.37, 2.17]	−0.02
Partner desires same number of children				
No	1.00	-	-	-
Yes	0.53	0.293	[0.18, 1.57]	−0.13
Has no partner	0.13	0.106	[0.03, 0.63]^‡^	−0.33
Don’t know	0.91	0.596	[0.25, 3.28]	−0.01

^*^ *p* < 0.001 ^**^ *p* = 0.003 ^§^ *p* = 0.009 ^‡^ *p* = 0.011 ^†^ *p* = 0.092; AOR = adjusted odds ratio

### Influences on family planning use among WLHIV

As Table 1 shows, the majority of Central and Eastern clients (92%) had used contraception in the previous months and reported they had not stopped using contraception (since recruitment). Contraceptive use was verified by clinical records at recruitment to the study. Of those using contraception, the single largest proportions (51% Central; 45% Eastern) were using short-term methods (pills and injectables). 34% Central and 27% Eastern clients were using condoms for family planning and 14% central clients and 27% Eastern clients were using LARC-PM including IUDs, implants and sterilisation – this represents just 16% WLHIV overall.

Of the women who were pregnant at some point during the study (*n* = 233) (i.e. while knowing they were HIV-positive) 24% reportedly became pregnant while using an FP method – all of whom used condoms or other short-term methods. Table 4 shows that of those using either condoms or other short-term methods as their main method of contraception 70% did not want another child. Of those women with three or more children the vast majority were using condoms or short-term methods.

**Table 4:** Fertility intention and parity by FP method use

Family planning method type			
	Short-term method *N* = 107 (%)*	Long-term method *N* = 33 (%)*	Condom *N* = 71 (%)*
Fertility Intention:			
Did not want another child	74 (69)	26 (79)	50 (70)
Wanted another child	33 (31)	7 (21)	21 (30)
Parity:			
1 child	13 (12)	6 (18)	13 (18)
2 children	41 (38)	10 (30)	26 (37)
3 children	27 (25)	10 (30)	19 (27)
>3 children	26 (24)	7 (21)	13 (18)

*Some percentages do not add up to 100% due to decimal points

Given these findings together with the very high desire among our participants to plan pregnancies or prevent them altogether, we particularly wanted to investigate what influenced the use of LARC-PM (defined as IUDs, implants and permanent methods), compared to short-term methods (defined as condoms, pills and injectables). All the variables in Table I were investigated for their influence on type of FP use. Only two variables were significantly associated with type of family planning method used at univariable level; Table 5 shows these results. The evidence is of mixed strength after co-adjusting in a multivariable model (columns 5–7). Being older than 25 years and being widowed/divorced/separated both significantly increase the odds of using LARC-PM, with age ultimately being the strongest predictor.

**Table 5:** Factors influencing type of family planning method used by WLHIV in Kenya

Factor	Short-term method, *N* = 179 (%)	Long-term method, *N* = 32 (%)	*χ* ^2^/Fisher’s *p-value*	Adjusted odds ratio	95% confidence interval	Standardized beta
Group						
Central Province	163 (86)	26 (14)	*0.114*	*Excluded*	*Excluded*	*Excluded*
Eastern Province	16 (73)	6 (27)				
Age						
16–25	15 (100)	0 (0)	*0.007*	1.00	-	-
26–35	100 (79)	27 (21)		7.06	[0.89, 55.93]^§^	0.48
36–45	64 (93)	5 (7)		1.67	[0.19, 14.98]^†^	0.12
Marital status						
Single	49 (82)	11 (18)	*0.007*	1.00	-	-
Married	112 (90)	12 (10)		0.93	[0.34, 2.52]^‡^	−0.02
Separated/Divorced/Widowed	18 (67)	9 (33)		3.59	[1.09, 11.88]^*^	0.22

Short-term methods were: condoms, pills and injectables; long-term methods were: IUDs, implants and permanent methods ^*^ *p* = 0.036, ^§^ *p* = 0.064, ^†^ *p* = 0.648, ^‡^ *p* = 0.886; Missing data in the two sub-categories means N is not equal to 240 for the outcome variable *type of family planning method* (missing = 29 observations)

### Exploring fertility intentions and motivators for contraception: Insights from qualitative data

Our quantitative data suggest a high number of unintended pregnancies among WLWH, despite a high contraceptive use coupled with a desire to stop having children. Our qualitative data help explore further such discrepancy and reveal additional reasons and factors that could affect women’s fertility intentions and motivate their preferences for contraception.

Table 6 shows the background socio-demographic variables for the qualitative participants, taken from the linked quantitative survey questionnaires.

**Table 6:** Socio-demographic characteristics of the qualitative study sample (*N* = 48)

	Study cohort	
Factor	Central Province *N* = 25 (%)	Eastern Province *N* = 23 (%)
Age		
16–25	1 (4)	3 (13)
26–35	15 (60)	19 (83)
36–45	9 (36)	1 (4)
Marital status		
Single	15 (60)	2 (9)
Married	10 (40)	21 (91)
Education		
Up to primary	23 (92)	19 (83)
Above primary	2 (8)	4 (17)
Employment		
Unemployed	6 (24)	16 (70)
Regular	18 (72)	6 (26)
Professional	1 (4)	1 (4)
Currently on ART		
No	5 (20)	8 (35)
Yes	20 (80)	15 (65)
Knowledge of partner’s HIV status		
No	8 (32)	6 (29)
Yes	17 (68)	15 (71)
Number of previous pregnancies		
Once	2 (8)	1 (4)
Two times	8 (32)	5 (22)
Three times	5 (20)	5 (22)
More than three times	10 (40)	12 (52)
Number of living children		
< 2 children	3 (12)	1 (4)
2 children	10 (40)	6 (26)
> 2 children	12 (48)	16 (70)
My desire for children affected when I tested HIV+		
No	7 (41)	7 (41)
Yes	10 (59)	10 (59)
Desire for children after testing HIV+		
Never have a child again	7 (70)	8 (80)
Have a child after careful planning	2 (20)	2 (20)
Quickly have another child	1 (10)	0 (0)
Disclosure of HIV status^a^		
Disclosed to partner	20	17
Disclosed only to other family member	0	4
Have not disclosed	3	2

NB*:* these data are taken from the linked-survey (quantitative data) for the qualitative sub-sample. There were missing data for some questions

^a^These data are from the qualitative interviews. Two participants in Central Province did not mentioned whether they disclosed or not

The majority of the participants were age 26–35. In Central Province (FP recruited) more women were single while in Eastern Province (PNC recruited) most were married. Across all clients the vast majority of women had only primary education or lower and were either unemployed or non-professionally employed. Almost all participants had at least two living children.

All women had tested HIV-positive at least 15 months before the interviews (since they had disclosed positive status to the researchers at recruitment). Across all clients, the majority of women were on ART and said they knew their partners’ status. There was significant missing data on HIV disclosure in the survey but this was revealed in the qualitative interviews – included in Table 6.

#### Influences on fertility intentions among WLHIV

Overall, the large majority of women interviewed said that they did not want (more) children after testing positive, even though only eight had already reached the number of children they had intended to have pre-diagnosis (typically four). Nevertheless, they thought it was important that all women had children and five women (all Central) explicitly said they wanted another child, but not immediately. All five were younger women with only one or two existing children.

Analysis of fertility intentions revealed a range of reasons why women wanted to avoid childbearing. This included financial difficulties, echoing the “breadwinner” analysis from the quantitative survey. Three further themes emerged which go beyond data available in the quantitative surveys: fear of the consequences of pregnancy, stigma related to breastfeeding decisions and the importance of planning safe pregnancies.

Most women mentioned financial difficulties as a very significant reason they wished to avoid having more children, unless pregnancies were carefully planned. Usually these difficulties arose from HIV related illness (of the women or their partners), death or separation (of the partner/breadwinner) which had a negative impact on caring for children:

*“I would not want to have another child because now I have three and am the one feeding them and if I add another one it will be a burden […] It was my wish I get the children while am with a husband […] but now the way it [HIV] came and we separated [after HIV diagnosis] you can see now it’s a burden.”* [Central Province, 3 children, separated, no current FP method]*.*

The fear of passing the virus on to an unborn baby was strong among all women:

*“I am afraid that I may infect the child.”* [Central Province, 2 children, married, condoms]

Equally powerful was the fear of the negative impact of a pregnancy on their health:

*“We were told that your blood continues to reduce, your body deteriorates badly. Now we were told that giving birth so much is not good.”* [Eastern Province, 3 children, married, injection]

The fear that pregnancy would worsen their own health was usually driven by a fear that if they died early they would leave their child(ren) orphaned so it was important to protect your own health in order to take care of your children.

Women in both cohorts mentioned that stigma affected their fertility decisions. One example of this is the dilemma women felt they faced over whether or not to breastfeed. Many women said they believed they would infect their child if they breastfed at all, but noted that if you avoided breastfeeding you would be stigmatised and labelled as HIV-positive:

*“[It] is a problem if you get a child and you do not breastfeed [Silence] … They [community] conclude for themselves… […] They just know you are positive”.* [Central Province, no children, has partner, condoms]

Therefore, some concluded it would be better not to have another child in order to avoid this dilemma. Many spoke of widespread stigma towards WLHIV having children at all:

*“They [people in the community] say, she [HIV positive woman] […] should do family planning not to continue giving birth to sick children”.* [Eastern Province, 3 children, married, condoms]

#### Good degree of understanding of the need to seek advice on how to bear children safely

As a result of the fears and difficulties described above most women were acutely aware of the need to either prevent or carefully plan a pregnancy – as one put it “*I don’t like getting a baby when I have not planned”*. Most had been given medical advice on reducing childbearing, planning or spacing children to minimise the negative health impact of pregnancy. Many women (including all those wanting another child) described how it was possible for HIV-positive women to have children safely (i.e. without infecting them) provided it was planned with a doctor’s advice:

*“there are ways that you will come and follow and the doctor will explain to you, you will be able to carry the pregnancy, you will be able to give birth, and prevent infecting the baby with the virus.”* [Central Province, 3 children, separated, no FP method]

In particular, women reported being advised by health care providers not to have more children while they had a weak immune system (measured by CD4 counts), but recognised that childbearing was still possible if planned for a time when immunity improved:

*“When the CD4 rises, we shall get a child.”* [Central Province,1 child, married, condoms]

*“[doctors] told […] if it [immunity] is too low and you want to give birth, that child will have the AIDS virus. [...] If your immunity is good, the doctor can allow you to get pregnant, but he will be checking on you as required until you give birth properly.”* [Eastern Province, 5 children, married, tubal ligation]

Nevertheless, one woman (with low education) emphasised the difficulties of the planning process itself and how this reduced her fertility intentions:

*“it will be a difficult process to get another child to go to the hospital again so that the child doesn’t contract the virus […] so maybe you prefer to only get two [children].”* [Central Province, 2 children, married, injection]

#### Contraceptive practices and motivations among WLHIV

In line with the quantitative data showing a high contraceptive use, interviews revealed a strong motivation to use contraception and high reported condom use both for FP and re-infection prevention. Several women reported using condoms only, after heavy promotion from their health providers, but many expressed anxiety that the condom might burst and put them at risk of unintended pregnancies. One woman had experienced this and became pregnant after her partner’s condom burst. Most women used short-medium term contraception in particular the injectable and the pill, although several expressed dissatisfaction with the pill (made them feel sick) or anxiety about forgetting to take the pill every day: for one woman this resulted in an unintended pregnancy. Several women also expressed dissatisfaction with injections (mainly because of heavy bleeding). Nearly one-third reported using dual protection (i.e. condoms in addition to another method) in the last six months in recognition that condoms were not always the best protection from pregnancy:

*‘We are using condoms and I still use that pill, because condoms prevent germs to enter into me and to him, but this pill for family-planning prevents that condom if it bursts I will get pregnant.’* [Central Province, 4 children, married, pill and condoms]

Nearly a quarter of the women interviewed (6 Central and 6 Eastern clients) reported using long-acting reversible contraceptives and permanent methods (LARC-PM): 6 used implants, 2 used IUD and 4 got sterilised. Several had experienced side-effects from other (short-term) methods but the primary motivation seemed to be effective prevention of unwanted pregnancies: all of them stated they did not want to have any more children and two of those who chose sterilisation had babies who died of HIV/AIDS. The majority of the women reporting using an implant were married, two were separated (one abused and one abandoned by their partner). Preferences for the implant were that it did not have side effects and it lasted a long time thus not requiring multiple hospital visits.

*“The one for three months is for a shorter period that is after every three months one has to go back, therefore the one for 5 years is cost effective and also saves time.”* [Eastern Province, 1 child, married, no post-partum FP use but wants Norplant]

*“Norplant is good, because….it has no side effects that may harm my body.”* [Central Province, 1 child, abandoned by partner, Norplant]*.*

The women who said that they got tubal ligation were all married and either with high parity (4–6 children) or had a baby who had died of HIV/AIDS. They said they got sterilised after the last pregnancy because they did not want to have any more children. Two of them said they were advised about tubal ligation by their doctor after their last baby. Only 2 women used the IUD. One woman who used the IUD said she chose it because she had problems with the injections. The other IUD user was advised by her doctor that it was an effective long-term method to prevent further pregnancies:

*“[…] you know when I gave birth I was told not to bear again ... now I decided to check this [coil] and let me put this one”.* [Eastern Province, 2 children, married, IUD]

For some, money was a barrier to FP use. Unlike anti-retroviral treatment contraception is not free to WLHIV in Kenya. One woman who had four children (of whom 3 were HIV+), did not want to have another but was not using FP remarked *“they told me to come with money”* – which she did not have. Several women said they wanted to use LARCs but were not supported to do so either by their partners or health providers.

Male partners were seldom involved in FP decisions, but over half of our participants said their partners knew their own (and their partner’s) status and in most of these cases high degrees of cooperation were reported for condom use:

*“he is happy to use [condoms] because he does not want us to give birth fast.”* [Central Province, 2 children, has partner, pill and condoms]

Nevertheless, many women faced male-related barriers to contraception use. For example, a number of women mentioned they could not use FP without their husband’s consent:

*“…if you want family planning ...you have to tell your husband ... if he tells you it is okay... you are going to plan”.* [Eastern Province, 5 children, married, Tubal ligation]

Women described how this consent might not be forthcoming for a whole range of reasons that are well documented in the family planning literature including male suspicion of family planning and expectations of controlling childbearing and contraceptive decision-making. One described how before she tested positive she was continuously childbearing: “*We got children following each other year after year”*. By the time she tested positive her husband’s desired number of children (three) had been reached and then he left it up to her to choose a family planning method:

*“…he said that I should use a family planning method. And I chose injection and I have never heard him with a problem with the injection.”* (Central Province, 3 children, married, injection and condoms]

Even when women reported partner refusal to use family planning, some of them said they tried to use it secretly, to avoid getting pregnant again against their will:

*“He was insisting I get another child […] so I saw if I don’t use family planning method I would get hurt by those children and also they would get hurt. He wanted me to get five children […] Now it was a must to use a method that didn’t require me to be going to the hospital because he would know. So it was a secret plan that I would use without his knowledge when I have that Norplant for five years he can’t know.”* [Central Province, 3 children, separated, no FP method since separation]

#### Limited medical advice on contraception

A few women reported receiving extensive information on FP from providers on the use (and dual use) of other FP methods, even facilitating FP switching if necessary, though this advice was rare:

*“There are women who can have side effects [from the pill]. I was told if I get side effects I go back for a check-up if there is injection.”* [Central Province, 1 child, single, IUD]

For most, they reported receiving little information from providers except about condoms. A number of women using condoms and short-term methods said they wanted to change to injections or norplant but had been told to wait (in two cases because they had recently given birth, but for others reasons were unclear):

*“I told them [I wanted] Norplant – they told me to wait.”* [Central Province, children (number not known), married, condoms]

## Discussion

### Policy and practice implications

#### Fertility desires and inappropriately met need for contraception

Our study of clinic users living with HIV found very high levels of contraceptive use. Nevertheless, for 40% of women their last (or current) pregnancy was unintended and 24% experienced this while using condoms or short-term family planning methods – which have been found elsewhere to be associated with greater risk of unintended pregnancies [24, 35]. Only 16% clients overall were using LARC-PM (IUDs, implants or permanent methods). One-third used condoms (as their main method) and all others used other short-term methods (pills and one- or three-monthly injectables).

Alongside this, almost three-quarters of women in our sample (both survey and in-depth interviews) expressed a desire for no further children. Our qualitative data confirmed other studies [36] showing WLHIV were fearful of unplanned pregnancies because of concerns over transmission of the virus to the unborn child, further damaging their own health status and financial worries. In the quantitative sample those who did want children in future had fewer existing children (less than two) and (a new finding) reliable breadwinners to support them and their children. The qualitative sample showed that despite most participants being educated below primary level they displayed a good degree of understanding of the need to seek advice on how to bear children safely and the critical need to prevent mistimed pregnancies and plan them at times when CD4 counts were good. This may not be surprising since they are clinic users, but in contrast to other studies that have found very weak information and support for WLHIV wanting to plan childbearing [37] our sample seemed to be confident they would get (or had already received) good advice. This bodes well for the achievement of UNAIDS’ target to reduce unmet need for FP and reduce mother-to-child-transmission to zero [21].

Effective contraception is understood as key to safe pregnancy-planning, but the qualitative data highlighted the difficult realities of using short-term or male-reliant methods that render them less effective for preventing unplanned pregnancies. In Kenya two qualitative studies, including one with 76 in-depth interviews more than half of whom were HIV-positive, have shown that gendered power imbalances and the negative views men hold of family planning significantly impede women’s consistent use of effective contraception, though some men were willing to accept condom use to avoid HIV-reinfection [38]. Combining all our evidence suggests that a substantial proportion of women may have an *inappropriately met contraceptive need* for their situation in which they may only – or mainly – be able to use condoms for family planning. Inconsistent use of condoms or pills or gaps in other short-term methods use can result in high rates of unintended or mistimed pregnancies that are potentially harmful to WLHIV and their unborn children.

#### Improving contraceptive and pregnancy-planning advice

Pregnancy is known to negatively affect WLHIV in a range of ways, particularly when CD4 counts are low [39–42], so there is a particular imperative to support women who wish to avoid or carefully plan pregnancy to do so effectively. Although not statistically significant, it is notable that 55–71% WLHIV in our survey received no FP advice from providers. The qualitative data reveal that on the rare occasions good advice was given and a choice offered, some of these women chose to use LARC-PM. Improving FP counselling that proactively implements WHO tier-effectiveness guidelines (in which condoms rate low) to avoid “misinformed choice” should be a priority [43]. In our sample some women had been given information on different contraceptive methods, though rarely on long-acting methods, and some had switched between methods at various times. One or two women had asked for LARC-PM and were very pleased to have a method which reduced the stress and anxiety of not being adequately protected from unplanned and harmful pregnancies.

Clearly condoms must continue to play a central role in HIV services, including for contraception. Short-term methods will also remain critically important for WLHIV trying to plan their fertility. But studies are beginning to emerge that see an important role for neglected long-acting methods for WLHIV in sub-Saharan Africa [10, 36, 44, 45] though few have explored the predictors and motivations for LARC-PM use. We found two highly significant predictors of using LARC-PM. First, women over the age of 25 years are more likely to be using LARC-PM, echoing the DHS data for all women which shows older women are more likely to use LARC-PM [25]. This may be because they are more likely to have achieved their desired fertility but it should be noted that the age is much younger in this WLHIV sample of clinic users (above 25 years) compared to the DHS where women above 35 years are more likely to use LARC and emphasis should be on expanding contraceptive choice to allow women to choose a method – either short or long-term – to suit her needs. Second, being widowed, divorced or separated is predictive which may be explained by a number of factors. It could be that women who were previously using LARC-PM while in a relationship but whose partner then died or left did not remove the method, though this is not substantiated in our qualitative data in which most women using LARC-PM were married. It may also reflect strong intentions among WLHIV who were previously in a long-term relationship to ensure no accidental pregnancy occurs when they feel they are not in a position to have a child (because they have no partner to provide support or are not in good health).

Expanding access to include LARC-PM may not be easy since these methods require a higher level of clinical skills and equipment than shorter-term methods; this has health systems, human resource and training implications. Commitment to service-integration seems likely to improve access for WLHIV: by providing LARC-PM alongside a choice of FP methods within specialist HIV/AIDS treatment centres or offering HIV care services within fully equipped mainstream family planning units. There is a huge, and growing, literature on integration of HIV and other reproductive health services but most does not explicitly deal with including LARC-PM as part of wider contraceptive choice for WLHIV. Nevertheless, there are many studies which show the benefits of improving contraceptive access to WLHIV through service-integration [46–48]. More powerfully, provision of free FP including LARCs for WLHIV may be the surest way of meeting fertility goals. A large study from the US found that when women were given a choice of free contraceptives at least 70% chose LARCs [49].

Understandably there is considerable sensitivity over advocating for the promotion of LARC-PM for WLHIV, given the hard-won commitment to non-discrimination and reproductive justice. There is no place for coercion in a rights-based family planning framework, and LARCs should be provided through programmes that are explicitly rights-based, ensure informed and voluntary choice, and provide discontinuation on demand [43, 47].

#### Measurement implications

If UNAIDS targets are to be met and WHO guidelines followed to meet the FP needs of WLHIV, facilities and providers need to do more to offer a wider and better informed choice of methods. Indeed, access to a wide range of effective contraception including LARC-PM is a recognised indicator in the measurement of the quality of family-planning services. In most standard DHS surveys, condoms are included under “modern methods”, even though they fall in WHO Tier 3 effectiveness. To allow measurement of “effective contraception” which facilitates “appropriately met need” condoms will need to be routinely separated as a method and short-term methods vs. LARC-PM also need to be routinely differentiated.

#### Limitations

Although for a mixed-methods study the sample sizes were good, some variables, including those that produce significance, attained only small numbers therefore further work with larger samples is needed to verify our predictive factors. More importantly, given the ethical challenges associated with collating data on HIV-status our sample of WLHIV were self-disclosing, not a random sample. Therefore there may be hidden characteristics that potentially bias our findings and the study should be treated as exploratory. Related to this, we did not have time-since-diagnosis data. Nevertheless, the triangulation between key findings from both the quantitative and qualitative findings do suggest that our core findings are valid and further work with larger random samples could confirm this. In the qualitative interviews, despite extensive discussion of contraceptive use, it was sometimes difficult to ascertain whether or not participants were utilising short-term methods, including condoms, consistently. There was little information on the influence of provider-related factors because these were rarely mentioned spontaneously and were not probed – however a systematic review of influences on fertility intentions among PLWHA did not find evidence of provider-level influences so this may not be an important omission [48]. Finally, the small number of women using LARC-PM means that further research with a larger sample is needed to understand the barriers to, and motivations for, use of LARC-PM by WLHIV, including the views of male partners on use of different kinds of contraception.

## Conclusion

The women living with HIV in our clinic-user sample have low fertility desires and a strong motivation to protect unborn and existing children by using contraceptives to prevent or plan pregnancies. Contraceptive use is high among these women but many had experienced an unintended pregnancy and many of these were using condoms or short-term methods at the time. This amounts to *inappropriately met contraceptive need* for many women, despite them being existing clients of reproductive health services. Improving access of WLHIV to a full range of FP counselling and contraceptives, including LARC-PM within a rights-based framework, would promote better informed choice, enable them to protect their fertility intentions, contribute to ensuring planned pregnancies and improve safe-child-bearing.
